# Cardan angle rotation sequence effects on first-metatarsophalangeal joint kinematics: implications for measuring hallux valgus deformity

**DOI:** 10.1186/1757-1146-7-29

**Published:** 2014-05-14

**Authors:** Ward M Glasoe, Fernando A Pena, Vandana Phadke

**Affiliations:** 1 Program in Physical Therapy, Medical School University of Minnesota, Mayo Mail Code 388, 420 Delaware St SE, Minneapolis, MN 55455, USA; 2 Orthopaedic Surgery, Medical School University of Minnesota, Riverside Campus, 2512 Seventh St. S, Minneapolis, MN 55454, USA

**Keywords:** Biomechanics measurement, Bunion deformity, Gait simulation

## Abstract

**Background:**

There currently are no recommended standards for reporting kinematics of the first-metatarsophalangeal joint. This study compared 2 different rotation sequences of Cardan angles, with implications for understanding the measurement of hallux valgus deformity.

**Methods:**

Thirty-one women (19 hallux valgus; 12 controls) participated. All were scanned in an open-upright magnetic resonance scanner, their foot posed to simulate the gait conditions of midstance, heel-off, and terminal stance. Using computer processes, selected tarsals were reconstructed into virtual bone models and embedded with principal-axes coordinate systems, from which the rotation matrix between the hallux and first metatarsal was decomposed into Cardan angles. Joint angles were then compared using a within factors (rotation sequence and gait condition) repeated-measures analysis of variance (ANOVA).

**Results:**

Only the transverse plane-first sequence consistently output incremental increases of dorsiflexion and abduction across gait events in both groups. There was an interaction (F ≥ 25.1; *p* < 0.001). Follow-up comparisons revealed angles were different (*p* < 0.05) at terminal stance.

**Conclusions:**

Different rotation sequences yield different results. Extracting the first rotation in the transverse plane allows for the resting alignment of the hallux to deviate from the sagittal plane. Therefore, representing first-metatarsophalangeal joint kinematics with the transverse plane-first rotation sequence may be preferred, especially in cases of hallux valgus deformity.

## Background

Three-dimensional kinematics are often calculated with Cardan (Euler) angle equations. Cardan angles are quantified as ordered rotations about 3 axes, and represent joint motion displacements in the anatomical body planes. Foot dorsiflexion/plantar flexion joint rotations occur about the mediolateral-axis in the sagittal plane; inversion/eversion about the longitudinal-axis in the frontal plane; adduction/abduction about the vertical axis in the transverse plane. To reduce the sequence dependent nature of the matrix calculation, the first rotation is typically extracted about the axis of largest joint motion and the last rotation about the long-axis of the distal segment [1]. Following these basic guidelines, the International Society of Biomechanics (ISB) recommended researchers adopt the sagittal, frontal, transverse rotation sequence as the standard for reporting kinematics of the ankle joint [2]. No standards have been recommended for the first-metatarsophalangeal (1-MTP) joint.

**Figure 1:**
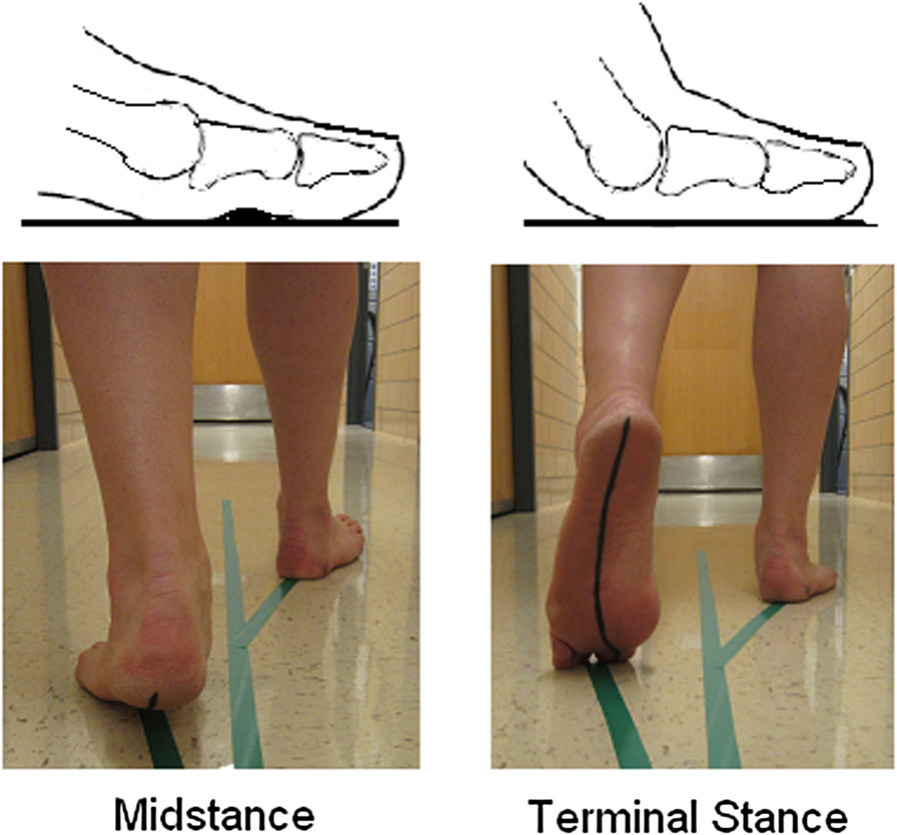
**The hallux behaves as the fulcrum point of gait propulsion.** Lines on the floor depict the foot-progression angle which averages 10° from the midsagittal line. The line on the foot estimates the normal pattern of center-of-pressure (COP) which shifts towards the hallux during the final stages of gait.

**Figure 2:**
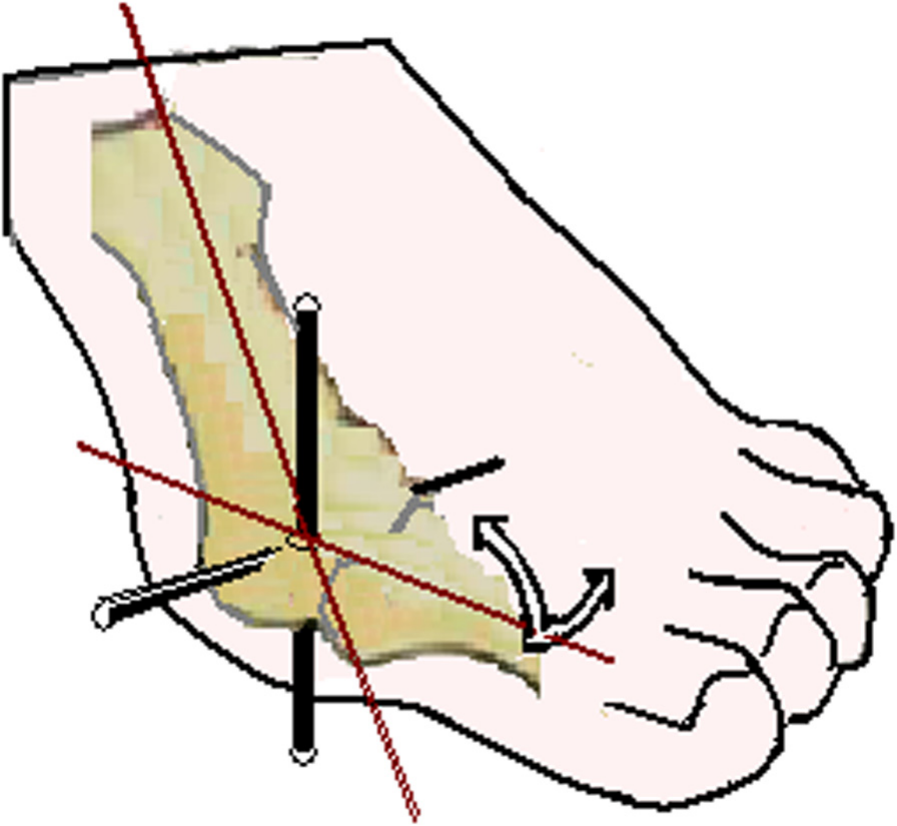
**Illustrates the biaxial joint system located in the first metatarsal.** Hallux dorsiflexion and plantar flexion occur about the horizontal-axis in the sagittal plane; abduction and adduction about the vertical-axis in the transverse plane. Dorsiflexion and abduction are the movements displayed with arrows.

The hallux plays an equally important role as the ankle during gait (Figure 1), and disorders involving the 1-MTP joint are common [3]. Stepping moves the hallux about a condyloid biaxial joint system (Figure 2) located in the first metatarsal head [4,5]. Orientation of the joint axes permit the hallux to rotate in the sagittal and transverse planes but acts to constrain the hallux from rotating independent of the first metatarsal in the frontal plane [4]. Aware of the anatomy, research describing 1-MTP joint with Cardan angles compute frontal plane (in/eversion) motion as the *last* order-of-rotation [6-11]. There is no consensus, however, on whether the *first* order-of-rotation should be computed in sagittal plane (dorsi/plantar flexion) [6,7] or the transverse plane (add/abduction) [10,11], especially in people with hallux valgus foot deformity [12].

Hallux valgus is chiefly characterized by an offset in transverse plane alignment of the 1-MTP joint. The hallux is abducted in relation to the long-axis orientation of the first metatarsal and when deformity is severe, abduction of the hallux exceeds 40 degrees [3]. Even absent of deformity, the resting posture of the hallux tends to align in some degree of abduction with respect to the first metatarsal and in relation to the midsagittal line of the body. To account for this offset in joint alignment, Glasoe et al. [10,11] modeled the 1-MTP joint using a transverse plane-first sequence. Other researchers [6,8,9] have followed ISB recommendations for the ankle, and modeled the 1-MTP joint using a sagittal plane-first sequence. Both solutions are correct, so preference becomes a matter of clinical interpretation.

The purpose of this study was to compare Cardan angle rotation sequence effects on 1-MTP joint kinematics during gait with implications for the measurement of hallux valgus deformity. Citing data recorded in gait trials [6,7], and premised on how the foot is placed during stepping (Figure 1) and the trajectory of body’s center of pressure which moves in response to ground reaction forces [13], dorsiflexion and abduction of the hallux should occur during heel rise. Due to the significance of the vertical force and because the collateral ligaments check motion of the hallux in the transverse plane, dorsiflexion should be largest. About 50 degrees of 1-MTP joint motion is needed for normal gait [6-8]. Deformity increases loading on the medial border of the hallux [3], so the direction of the respective external and internal foot-to-ground contact forces may reduce dorsiflexion while further encouraging abduction of the hallux.

In summary, this study compared “sagittal” and “transverse” plane-first rotation sequence effects of 1-MTP joint angles in women grouped based on the presence or absence of hallux valgus deformity. The data were analyzed under 2 hypotheses. First, that different Cardan angle rotation sequences would output different angles. Second, that both rotation sequences would output increasing amounts of hallux dorsiflexion and abduction across gait events regardless of group assignment.

## Methods

### Participants

The experiment was retrospective in design. The data were collected on 31 (of 31 total) adult women enrolled in a two-part investigation [10,11] that used weightbearing imaging to study tarsal kinematics. A comprehensive description of the participants and research methods has been reported elsewhere [10,11]. Nineteen of the women had hallux valgus. Measurement (on images) of a hallux angle larger than 15° served as the threshold for classifying deformity. The hallux angle quantifies the offset in transverse plane alignment of the 1-MTP joint. The hallux was abducted 33° (range, 18 to 64°) with deformity, compared to 8° (range, -2 to 14°) in controls. Participants were group matched for demographics: age 48 ± 17 years; BMI 26 ± 6 kg/m^2^. All gave informed consent in accordance with University of Minnesota Human Research Institutional Review Board (#0709 M16823) guidelines.

### Experimental procedures

**Figure 3:**
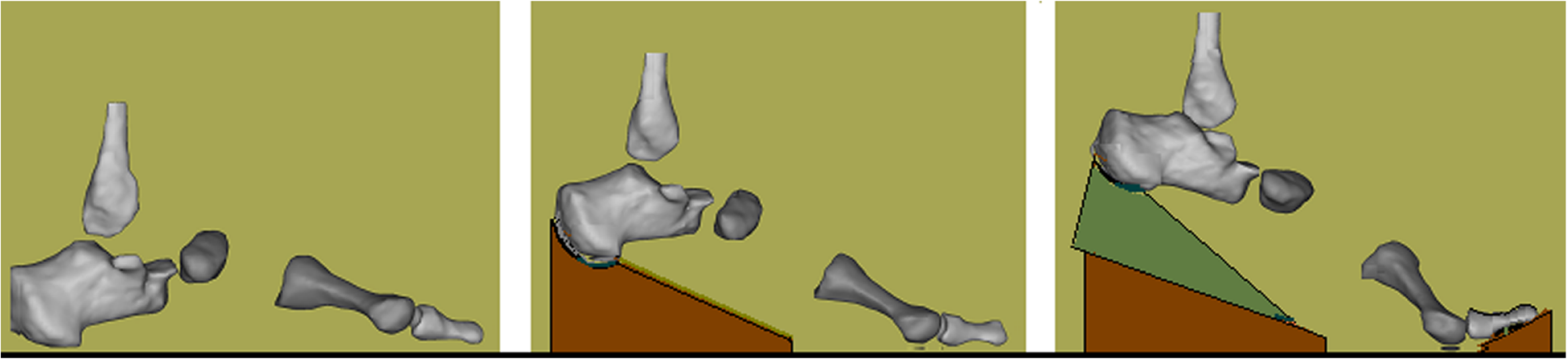
**Virtual bone models.** Sagittal view of the bones reconstructed from magnetic resonance (MR) images. The foot was posed on a system on wedges to simulate the gait midstance (MS), heel off (HO), and terminal stance (TS). The bones are the proximal phalanx of the hallux, first metatarsal, navicular, calcaneus, and distal end of the fibula.

Participants were imaged in an open-upright 0.6 Tesla MR scanner, their foot posed to simulate gait midstance (MS), heel off (HO) and terminal stance (TS) [10,11]. Gait was simulated by placing the foot on identical seized wedges (Figure 3). No wedging was used at MS. One wedge having a 15° angle was placed beneath the calcanei at HO. Two wedges were stacked and placed beneath the calcanei bilaterally at TS. A wedge angled at 10° was also placed beneath the toes at TS (Figure 3) to tighten the plantar fascia [10,11].

Following imaging, selected bones (Figure 3) including the proximal phalanx of the hallux and the first metatarsal were reconstructed and registered across the gait conditions within the MR laboratory frame-of-reference system using MIMICS (Materialize, Leuven, Belgium). Principal component inertial-axes systems were embedded to define the spatial orientation of each virtual bone model [14]. Coordinate system axes were directed Z-lateral (dorsi/plantar flexion), the Y-up (add/abduction), and X-anterior (in/eversion). The coordinate systems defining the hallux and first metatarsal embedded consistently and aligned nearly coincident with the MR frame of reference. The error associated with data processing was < 3 degrees [15]. The measurement of tarsal motion was shown reliable and valid across the simulated gait conditions [10,11].

### Data analysis

First metatarsophalangeal joint angles were extracted from two different transformation matrices calculated from one Matlab (Mathworks Inc, Natick, Massachusetts) code. The sagittal plane-first (Z-Y-X) sequence followed ISB guidelines for the ankle joint [6,8,9], while the transverse plane-first (Y-Z-X) sequence was thought to better represent kinematics of the 1-MTP joint [12]. Joint angles were described as mean values across gait conditions, and examined using a 2-way within factors (rotation sequence and gait condition) repeated measure analysis of variance (ANOVA). In the presence of an interaction, follow-up pairwise comparisons were completed at each level of condition. The ANOVA model was run separately on the two groups (hallux valgus and controls). Significance was set at *p* < 0.05.

## Results

**Figure 4:**
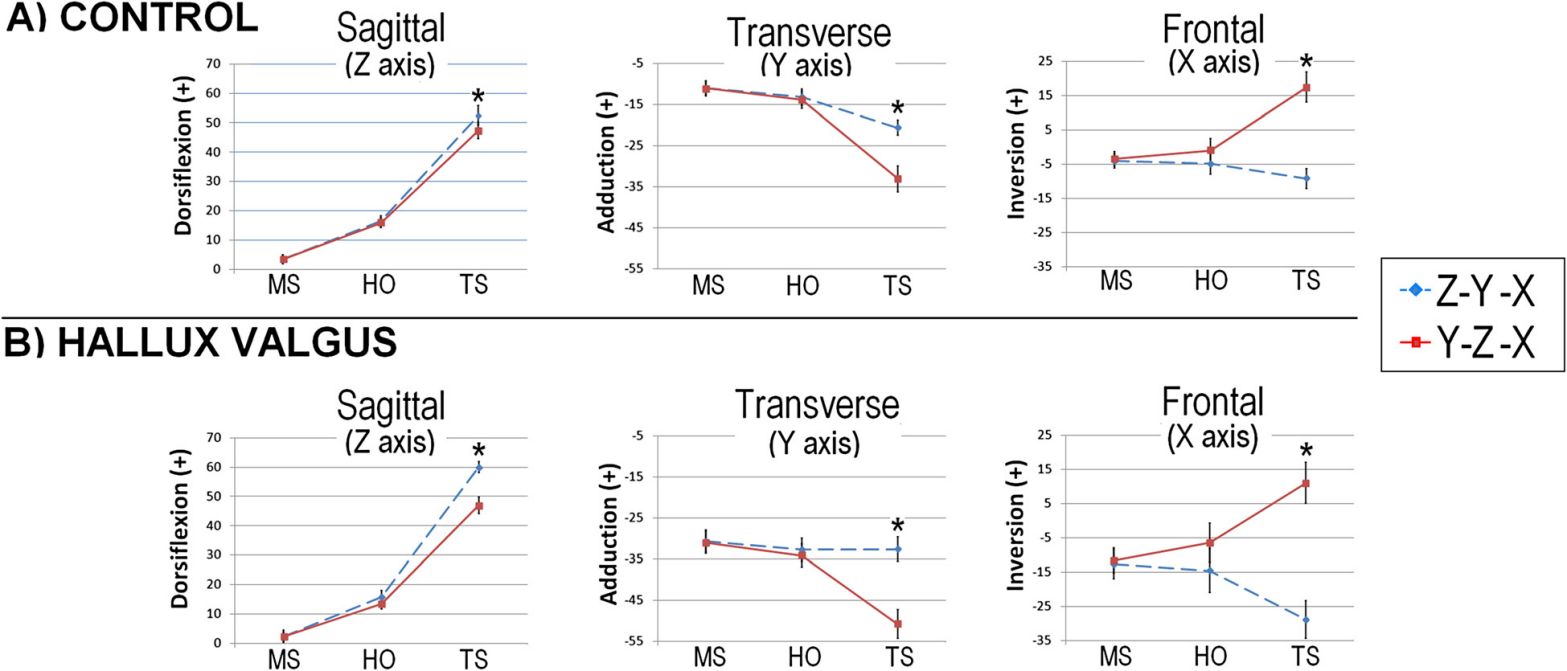
**First metatarsophalangeal joint angles plotted across gait conditions. A)** Angles for the control group. **B)** Angles for the hallux valgus group. Error bars are 95% confidence intervals. Asterisks (*) indicate difference (*p* < 0.05) in pairwise comparison between sequence within conditions. Note the scale change between graphs. Abbreviations: MS, midstance; HO, heel off; TS, terminal stance.

Figure 4 displays 1-MTP joint angles processed from the 2 rotation sequences. Regardless of group or sequence, dorsiflexion increased across gait conditions. Dorsiflexion increased by an average of 15° from MS to HO, and about 35° from MS to TS. Only the transverse plane-first rotation sequence consistently output incremental increases of abduction. Abduction increased an average of 5° from MS to TS, and about 20° from HO to TS in both groups when angles were represented with the transverse-first sequence, but not when represented with the sagittal plane-first sequence (Figure 4).

There was an interaction (F ≥ 25.1; *p* < 0.001) between variables. The follow-up comparisons revealed all mean angles were different (*p* < 0.05) at TS. Differences were ≥ 5.1° in the sagittal plane about the Z-axis, ≥ 12.5° in the transverse plane about the Y-axis, and ≥ 8.2° in the frontal plane about the X-axis. The frontal plane angles were also divergent in direction.

## Discussion

De Mits et al. [6] and Leardini et al. [7] used the sagittal plane-first sequence to model 1-MTP joint with Cardan angles on adults without foot deformity. Dorsiflexion and abduction started before midstance and continued unabated until the hallux lifted off the ground at the last instant of push-off. Dorsiflexion was dominant in both studies. De Mitts [6] recorded 55 degrees of dorsiflexion and 50 degrees of abduction. Leardini [7] recorded 40 degrees of dorsiflexion and 10 degrees of abduction. Although the difference in joint motion between trials is notable, these results indicate that dorsiflexion and abduction should occur at the 1-MTP joint into late stance phase of gait (Figures 1 and 2). Consistent with data reported in these past gait trials [6,7], increasing amounts of dorsiflexion and abduction were expected in this study - where gait was simulated by posing the foot on a system of wedges (Figure 3). When interpreting these results, it is important to remember the participant’s foot was positioned the same for both sequences, so any difference in 1-MTP joint angles shows the effects of the rotation sequence and nothing else.

Regardless of group assignment, both sequences captured increasing dorsiflexion (Figure 4) as should happen during gait (Figure 1). Dorsiflexion surpassed 50 degrees, with 15 degrees the increment of increase of from MS to HO, and 35 degrees from HO to TS. This total amount as well as the incremental change in dorsiflexion between gait conditions closely approximate the angle of wedging used to advance the foot to simulate gait (Figure 3).

Transverse plane abduction was the next largest component of motion (Figure 4). Of the 2 tested sequences, only the transverse plane-first sequence (Y-Z-X) output increasing abduction (~20°) across gait conditions in both groups. The sagittal plane-first sequence, in comparison, output as abduction (<5°) from MS to HO, and adduction (<5°) from HO to TS in the group with hallux valgus. Based on the placement of the foot, it is doubtful the hallux could *adduct* while pressed into the ground as the heel lifts and weight advances forward and displacement in the direction of adduction becomes even more unlikely in the group having hallux valgus deformity. Although the kinetics were not simulated, terminal stance gait propulsion has an active component powered by the muscles that flex the toes and ankle, and a passive component attributed to the tightening of the plantar fascia [16]. As a consequence of hallux valgus deformity, pull of the flexor muscles and plantar fascia acting on the hallux shift to the lateral side of the 1-MTP joint. Loading must therefore abduct the hallux, but meaningful abduction (~20°) was represented in those with hallux valgus only when the 1-MTP joint angles were modeled with the transverse plane-first rotation sequence.

This implausible Cardan angle group result merits further explanation. The greater disparity between sequences in the hallux valgus group (Figure 4) is due to greater deviation in initial resting alignment of the 1-MTP from the straight (sagittal plane) dorsi/plantar flexion axis. Because resting posture alignment is not even close to straight (hallux angle = 33°) with hallux valgus, use of the sagittal plane-first sequence results in the first rotation occurring about a non-physiologic joint axis, whereas use of the transverse plane-first sequence adjusts for the initial presence of the abduction deformity, and then records dorsi/plantar flexion about that position.

The frontal plane angles were calculated as the *last* order-of-rotation. Divergent in direction (Figure 4), this difference in 1-MTP joint angles best highlight the sequence dependency effects of the Cardan angle calculation. Compounding the challenge of modeling the loading behaviors of the hallux in the frontal plane, surface palpation as required by most motion analysis techniques concentrates error around the long-axis of the hallux and first metatarsal [17]. Researchers choosing to calculate the frontal plane position of the 1-MTP joint as the *last* rotation should exercise caution when interpreting this result.

This retrospectively designed study had limitations. Gait was simulated with static conditions. Posing a person in a MR scanner does not replicate the loading demands of gait. Importantly, joint angles were not validated by other methods of direct measurement, though the computation of 1-MTP joint dorsiflexion did approximate the angle of wedging used to advance the foot in staging gait. The study was also narrow in scope, as it singularly compared the computation of 1-MTP joint motion using the 2 most common rotation sequences. This line of research could be broadened to compare possible matrix multiplication differences associated 3D helical axis computations, or projection errors associated with direction cosine. In light of these limitations, this study does not settle the issue but demonstrates why basic guidelines that govern order-of-rotation decisions at other joints may not apply to the 1-MTP joint, as the hallux typically rests in a position of relative abduction.

## Conclusions

The rotation matrices describing the Cardan angles between the first metatarsal and hallux were decomposed for the purpose of comparing sagittal plane-first and transverse plane-first rotation sequence effects. Both sequences output increasing amounts of dorsiflexion, only the transverse plane-first rotation sequence consistently output increasing amounts of abduction, and angles of in/eversion were divergent when representing 1-MTP joint displacements across gait conditions. Representing 1-MTP joint kinematics with a transverse, sagittal, and frontal plane sequence (Y-Z-X) may be preferred, especially in cases of hallux valgus deformity.

## Competing interests

The authors have no financial or personal conflicts that could influence the submitted work.

## Authors’ contributions

WMG and FAP provided the concept and research design, and secured the funding support. WMG and VP performed data collection and data analysis. WMG wrote the manuscript while the other authors provided critical review. All authors read and approved the final manuscript.
